# The Impact of Left Atrial Size in Catheter Ablation of Atrial Fibrillation Using Remote Magnetic Navigation

**DOI:** 10.1155/2018/3096261

**Published:** 2018-12-05

**Authors:** Xiao-yu Liu, Hai-feng Shi, Jie Zheng, Ku-lin Li, Xiao-xi Zhao, Shi-peng Dang, Ying Wu, Yan Cheng, Xiao-yan Li, Zhi-ming Yu, Ru-xing Wang

**Affiliations:** ^1^Department of Cardiology, Wuxi People's Hospital affiliated to Nanjing Medical University, Wuxi 214023, China; ^2^Department of Cardiology, Beijing Hospital, Beijing 100005, China

## Abstract

**Objective:**

The objective of this study was to investigate the impact of left atrial (LA) size for the ablation of atrial fibrillation (AF) using remote magnetic navigation (RMN).

**Methods:**

A total of 165 patients with AF who underwent catheter ablation using RMN were included. The patients were divided into two groups based on LA diameter. Eighty-three patients had small LA (diameter <40 mm; Group A), and 82 patients had a large LA (diameter ≥40 mm; Group B).

**Results:**

During mapping and ablation, X-ray time (37.0 (99.0) s vs. 12 (30.1) s, *P* < 0.001) and X-ray dose (1.4 (2.7) gy·cm^2^ vs. 0.7 (2.1) gy·cm^2^, *P*=0.013) were significantly higher in Group A. No serious complications occurred in any of the patients. There was no statistical difference in the rate of first anatomical attempt of pulmonary vein isolation between the two groups (71.1% vs. 57.3%, *P*=0.065). However, compared with Group B, the rate of sinus rhythm was higher (77.1% vs. 58.5%, *P* < 0.001) during the follow-up period. More patients in Group A required a sheath adjustment (47/83 vs. 21/82, *P* < 0.001), presumably due to less magnets positioned outside of the sheath. *In vitro* experiments with the RMN catheter demonstrated that only one magnet exposed created the sheath affects which influenced the flexibility of the catheter.

**Conclusions:**

AF ablation using RMN is safe and effective in both small and large LA patients. Patients with small LA may pose a greater difficulty when using RMN which may be attributed to the fewer magnets beyond the sheath. As a result, the exposure of radiation was increased. This study found that having at least two magnets of the catheter positioned outside of the sheath can ensure an appropriate flexibility of the catheter.

## 1. Introduction

Remote magnetic navigation (RMN) has been widely used for atrial fibrillation (AF) ablation [1–6]. Compared with manual ablation, it has the advantages of less complications and X-ray exposure [1–6]. However, because it was cost prohibitive, the RMN system was not used widely in China; therefore, the application experience is limited. In 2013, Wuxi People's Hospital began to use RMN for AF ablation due to the advantages reported in the literature. By the end of 2017, more than 200 procedures had been completed, thus increasing this center's expertise with the RMN technology. During this experience, it was found that the left atrial (LA) size impacted the mapping and ablation procedure, and the reasonable hypothesis was that the number of magnets outside the sheath may play a role in the navigation in small LA. The aims of this study were to discuss the impact of different LA size on AF ablation using RMN and the methods to overcome this problem.

## 2. Materials and Methods

### 2.1. Study Population

From March 2014 to January 2018, a total of 165 patients with AF who underwent ablation therapy for first time using RMN in Wuxi People's Hospital were included in this analysis. Due to the good efficacy and safety, RMN ablation was performed in all AF ablation cases in our center. The baseline characteristics of patients are listed in Table 1. All patients failed at least one antiarrhythmic drug. Routine blood, liver and kidney function, coagulation index, electrocardiogram, and echocardiography were performed for each patient before operation, and esophageal echocardiography was performed 24 hours before operation to exclude LA thrombus. Patients were on either warfarin or dabigatran for anticoagulation therapy and were discontinued three days before the procedure and bridged with low molecular heparin according to the patient's weight. For patients taking warfarin, after 12 hours of the procedure, warfarin was administered orally until the INR was greater than 2.0, and then, the low molecular heparin was stopped. For patients taking dabigatran, low molecular heparin and dabigatran were given 12 h after the procedure simultaneously, and then, the low molecular heparin was discontinued. All patients signed informed consent before the procedure. The patients were divided into two groups based on echocardiographic LA size according to the recommendations of the American Society of Echocardiography and the European Association of Cardiovascular Imaging [7]. Group A was defined as the LA diameter <40 mm, and Group B was defined as the LA diameter ≥40 mm [7].

### 2.2. AF Ablation Using RMN

Two electrophysiological study catheters were positioned: a 6F quadrupole catheter (Inquiry, St. Jude Medical Inc., St. Paul, MN, USA) at the right ventricular apex and a 6F decapolar coronary sinus catheter (Inquiry, St. Jude Medical Inc., St. Paul, MN, USA) in the coronary sinus via the femoral vein. After a fluoroscopically guided single transseptal puncture, an SR0 sheath (St. Jude Medical Inc., St Paul, MN, USA) was advanced into the LA. After transseptal catheterization, intravenous heparin was injected 50∼100 IU/kg and administered to maintain an activated clotting time of 250 to 350 seconds. A magnetic mapping and ablation catheter (Navistar Thermocool RMT, Biosense Webster Inc., Diamond Bar, CA, USA) was advanced through the sheath into the LA.

Carto RMT (Biosense Webster Inc., Diamond Bar, CA, USA) was used in conjunction with the RMN Niobe® II system (Stereotaxis, Inc., St. Louis, MO, USA) to perform stepwise remote-controlled magnetic 3D LA electroanatomic mapping. The Carto system transfers real-time catheter tip location and orientation to the RMN system, and the RMN system controls the direction of the catheter. The catheter-advancing system (QuikCAS®, Stereotaxis Inc., St. Louis, MO, USA) controls the advancement and retraction of the catheter. The voltage model of the LA was constructed under bipolar voltage mapping.

Radiofrequency ablation was performed in a temperature-controlled model. Radiofrequency delivered with a target temperature of 43-degree celsius. Power was limited to 35 W at the anterior LA wall and 30 W at the posterior LA wall with the irrigation rate set to 17 ml/min. Patients with paroxysmal AF were undergoing circumferential pulmonary vein (PV) ablation only. For patients with nonparoxysmal AF, a roof line and a mitral isthmus line ablation were performed. Radiofrequency current was applied for up to 30–60 seconds or until the maximal local electrogram amplitude reduced by 80%. The endpoint of the ablation was defined as bidirectional conduction block. This was verified by careful application of the Lasso electrode (Biosense Webster Inc., Diamond Bar, CA, USA) around the entire circumference of the PV ostia and pacing within the circumferential line at multiple sites. After the first anatomical attempt, in case of residual potentials at any location in the ablation line, a gap was suspected, and an additional ablation lesion was created.

### 2.3. Follow-up

All patients were followed at monthly visits for at least 6 months. All patients continued antiarrhythmic therapy at least 2 months. Holter monitoring was performed if necessary (symptoms of palpitation, etc.) in our hospital. In this study, after the three-month blanking period, the recurrence of AF was defined as atrial fibrillation documented by electrocardiogram or Holter examination. Patients were considered a success if they remained in sinus rhythm from the 3-month postoperative period to the end of the follow-up, with no evidence of AF. If the patient experienced AF during the blanking period, but no evidence of AF was found after three months from the ablation, then they were considered a success. Antiarrhythmic drugs were terminated; otherwise, they would continue in patients without freedom from AF. Two patients underwent reablation, but they were not included in this study.

### 2.4. Complications

Complications were divided into two categories: serious and minor. Serious complications included acute myocardial infarction, stroke, major bleeding, PV stenosis, LA esophageal fistula, and pericardial effusion/cardiac tamponade. Minor complications included pericarditis and inguinal haematoma.

### 2.5. Adjunctive *in Vitro* Experiments

**Figure 1:**
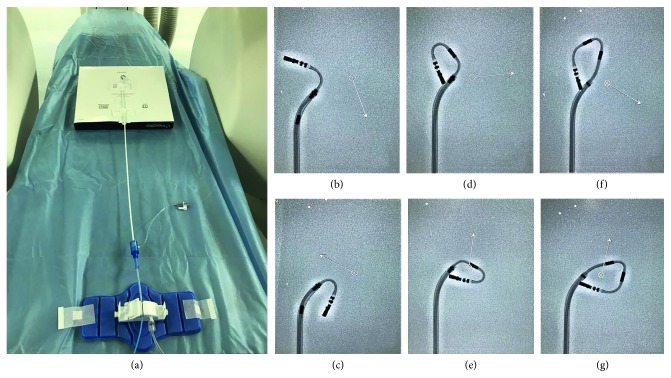
*In vitro* experiments showed the effect of different amounts of magnets deployed beyond the sheath tip on the flexibility of the catheter. (a) The work position of the RMN, catheter, and SR0 sheath. (b) and (c) The maximum catheter reach in two different orientations with one magnet deployed. (d) and (e) The maximum catheter reach in two different orientations of two magnets deployed. (f) and (g) The maximum catheter reach in two different orientations with all three magnets deployed.

It was hypothesized that the number of magnets outside the sheath may play a role in the navigation in small LA. Therefore, *in vitro* experiments were designed to determine how the number of magnets beyond the sheath affects the flexibility of the RMN catheter.  Figure 1 demonstrates the *in vitro* model of the RMN system simulating through a fixed SR0 sheath and varying catheter positions. Using the QuikCAS catheter-advancing system to control the advancement and retraction of the catheter, three different physicians controlled the RMN system at least three times, to confirm the maximum deflection of the catheter. This was performed with one magnet, two magnets, and all the magnets outside the sheath, and the maximum movement of the catheter was observed and recorded by X-ray.

### 2.6. Statistical Analysis

SPSS v19.0 statistical software was used for analysis. Continuous variables with normal distribution were expressed as mean±standard deviation. Continuous variables without normal distribution were reported as median (interquartile range). Categorical variable is expressed as a percentage. The independent sample Student's *t* test was performed for comparison of normal distribution variables, and the nonparametric test (Mann–Whitney) was performed for nonnormal distribution variables. The comparison of rates was confirmed by the chi-squared test. Age, gender, BMI <24 kg/m^2^, paroxysmal AF, LA diameter <40 mm, basic diseases, without four classical PV ostia patterns, procedure duration ≥141.6 min, duration from mapping begin to ablation finish ≥98.0 min, and total X-ray time ≥432.0 s were used for multivariate analysis with logistic regression to estimate if these parameters are associated with the using of X-ray during mapping and ablation. The Kaplan–Meier survival function was used to analyze the event-free survival rate, and differences between groups were assessed using the logrank test. *P* value <0.05 was considered to be statistically significant.

## 3. Results

### 3.1. Baseline Patients' Characteristics

Baseline patients' characteristics are shown in Table 1. No statistical differences were found between the two groups of patients for gender, age, height, AF duration, left ventricular ejection fraction, the proportion of diabetes, coronary heart disease, and stroke, and four classical PV ostia patterns (*P* > 0.05). Compared with Group B, body weight, body mass index, the proportion of hypertension, and the proportion of nonparoxysmal AF were lower in Group A (*P* < 0.05).

### 3.2. Ablation Procedure Data

Ablation procedure data are shown in Table 2. There were no statistical differences in procedure duration and total X-ray time between the two groups (*P* > 0.05). However, the total X-ray dose was higher in Group B (*P* < 0.001). The X-ray time and dose during mapping and ablation of Group A patients were significantly higher than those in Group B (37.0 (99.0) s vs. 12.0 (30.1) s, *P* < 0.001, and 1.4 (2.7) gy·cm^2^ vs. 0.7 (2.1) gy·cm^2^, *P*=0.013). More patients in Group A (80/83 patients) used X-ray guidance during mapping and ablation than Group B (68/82 patients) (*P*=0.01). In Group A, 47/83 of the patients had the SR0 sheath adjustment at least once, and 13/83 patients needed the SR0 sheath adjusted multiple times. In Group B patients, only 21/82 patients had a single SR0 sheath adjustment, and only 2/82 patients had the SR0 sheath adjusted more than one time. No serious complications occurred in either group.

### 3.3. Outcomes and Follow-up Data

**Figure 2:**
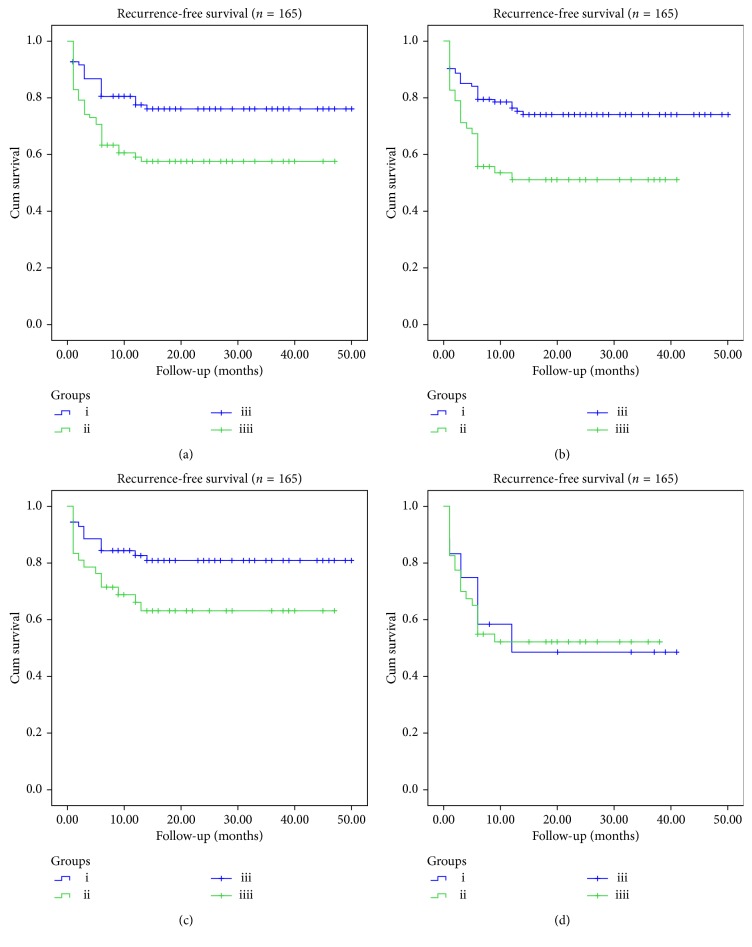
Kaplan–Meier curves of recurrence-free survival after primary successful ablation. (a) Kaplan–Meier curves demonstrate recurrence-free survival for patients with small left atrial (LA) group and large LA group. (i) Patients with LA diameter <40 mm, (ii) patients with LA diameter ≥40 mm, (iii) patients with LA diameter <40 mm censored, and (iv) patients with LA diameter ≥40 mm censored. (b) Kaplan–Meier curves demonstrate recurrence-free survival for patients with paroxysmal atrial fibrillation (AF) group and nonparoxysmal AF group. (i) Patients with paroxysmal atrial fibrillation, (ii) patients with nonparoxysmal atrial fibrillation, (iii) patients with paroxysmal atrial fibrillation censored, and (iv) patients with nonparoxysmal atrial fibrillation censored. (c) Kaplan–Meier curves demonstrate recurrence-free survival for patients with paroxysmal AF in small LA group and large LA group. (i) Paroxysmal atrial fibrillation patients with LA diameter <40 mm, (ii) paroxysmal atrial fibrillation patients with LA diameter ≥40 mm, (iii) paroxysmal atrial fibrillation patients with LA diameter <40 mm censored, and (iv) paroxysmal atrial fibrillation patients with LA diameter ≥40 mm censored. (d) Kaplan–Meier curves demonstrate recurrence-free survival for patients with nonparoxysmal AF in small LA group and large LA group. (i) Nonparoxysmal atrial fibrillation patients with LA diameter <40 mm, (ii) nonparoxysmal atrial fibrillation patients with LA diameter ≥40 mm, (iii) nonparoxysmal atrial fibrillation patients with LA diameter <40 mm censored, and (iv) nonparoxysmal atrial fibrillation patients with LA diameter ≥40 mm censored.

Outcomes and follow-up data are shown in Table 3 and Figure 2. The total rate of first anatomical attempt of PV isolation is 64.2% with no statistical difference found between the two groups (71.1% vs. 57.3%, *P*=0.065). However, the success rate of first anatomical attempt in paroxysmal AF patients was higher than nonparoxysmal AF patients (73.5% vs. 44.2%, *P* < 0.001). By June 2018, the average follow-up time of the patients in this study was 24.0 (20.0) months. Compared with Group B, patients in Group A had lower recurrence rate, and a higher proportion of patients remained in sinus rhythm (*P*=0.009). Subgroup analysis showed that the recurrence rate of paroxysmal AF in Group A was lower than that in Group B (*P*=0.036), but the recurrence rate of patients with nonparoxysmal AF in Group A was similar to that in Group B (*P*=0.978). From another perspective, patients with paroxysmal AF have lower recurrence rate than patients with nonparoxysmal AF (24.8% vs. 48.1%, *P*=0.002).

### 3.4. Logistic Regression Analysis

The results of logistic regression analysis are shown in Table 4. Age, gender, BMI <24 kg/m^2^, paroxysmal AF, LA diameter <40 mm, basic diseases, without four classical PV ostia patterns, procedure duration ≥141.6 min, duration from mapping begin to ablation finish ≥98.0 min, and total X-ray time ≥432.0 s were analyzed to determine if they were predictors of X-ray application for mapping and ablation. According to the result of logistic regression analysis, only LA diameter <40 mm proved to be an independent predictor in multivariate analysis associated with the use of X-ray during mapping and ablation (OR, 7.3; 95% CI, 1.7–31.0).

### 3.5. *In Vitro* Experiments

Figures 1–1 illustrate the sheath position, the catheter deflection, reach, pivot position, and curve shape in each RMT catheter relative to the catheter length deployed from sheath. The flexibility of the catheter was represented by the distance between catheter tip and sheath. To demonstrate the catheter flexibility, attempts were made to touch the distal tip of the catheter to the sheath by moving the tip inferiorly. Attempts to touch the sheath with one magnet positioned outside of the sheath were not possible; however, when 2 or 3 magnets were positioned outside of the sheath, the catheter tip could reach the sheath. This experiment suggests that having one magnet outside the sheath limits the flexibility of the catheter.

## 4. Discussion

This is the first study focusing on the impact of LA size in AF ablation using RMN. Our results indicate that the small LA may bring challenges in navigating the LA using RMN; however, this can overcome with sheath maneuvers that allow at least two magnets to be exposed but at the cost of an increase in X-ray exposure. *In vitro* experiments confirmed that with only one magnet deployed, the flexibility of the catheter is reduced. Despite the limitations, patients in our study with small LA had lower recurrence rate and the higher rate of sinus rhythm in follow-up. There were no serious complications reported in either group; thus, the additional sheath adjustments did not affect the safety profile in this study.

AF ablation using RMN is safe and effective [1–6]. Previous studies have confirmed that the RMN system can provide more accurate and stable catheter tissue contact, potentially increasing the efficiency of the ablation [8]. However, many studies have shown that the success rate of AF ablation using RMN is not higher than that of manual procedure, and the recurrence rate is similar during period of 6 to 18 months follow-up [2, 3, 5, 6, 9]. Further studies reported that the RMN system has unique advantages. Firstly, using RMN can obviously reduce severe complications such as death and cardiac tamponade [1, 5, 10]. Secondly, RMN ablation can significantly reduce the X-ray time and dose of patients and operators [1, 5, 10]. However, because it was cost prohibitive, the RMN system was not used widely in China; therefore, the application experience is limited.

As found in other reports, this study has shown that mapping and ablation of AF using RMN is not only safe and effective but also reduces the rate of complications and the radiation exposure [6]. Similar results are shown in this study, despite challenges of navigating the RMT catheter in smaller LAs. This was especially true in some locations such as the lower right PV, where the catheter during mapping and ablation point became unstable, and the tip of the catheter often jumped away or had difficulty during movements when the magnetic field direction was adjusted. In this study, the data of patients with the LA diameter <40 mm were analyzed and compared with patients with the LA diameter ≥40 mm. The results showed that during mapping and ablation, the catheter was more challenging to navigate in a small LA. However, this was remedied by making sheath adjustments under X-ray guidance to ensure the flexibility of the ablation catheter. This results in a significant increase in X-ray time and dose during mapping and ablation and was only a small portion of the overall dose; however, it did not affect the overall X-ray time for the procedure, which was slightly longer for Group B patients. Due to the differences in sheath maneuvers between the two groups, we hypothesized that the flexibility of the catheter may be compromised; therefore, the *in vitro* experiments were designed to determine how the number of magnets beyond the sheath affects the flexibility of the RMN catheter.

The RMN system is designed to steer the magnetic catheter by the three magnets located in the flexible distal portion of the catheter. The combined adjustments of the catheter length and the magnetic field direction are used to make precise movements and establish stable focal contact. This unique characteristic allows the physician to maintain control of the electrode position independent of the complexity of the path. However, during the procedure in the patients with a small LA, the operation space of magnetic catheter is also limited, thus reducing the flexibility of catheter, inducing the instability of catheter, and increasing the difficulty in navigation.

The logistic regression analysis showed that only LA diameter <40 mm proved to be an independent predictor associated with the using of X-ray during mapping and ablation, while other factors, including the variation of PV anatomy, were not related. This suggests that other factors did not increase the difficulty of RMN catheter operation.

**Figure 3:**
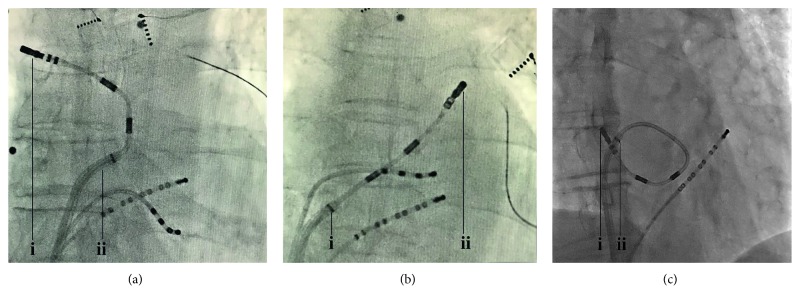
Fluoroscopic views in anteroposterior projection showing the special position of the sheath and RMT catheter. (a) The sheath (ii) points to the left and the catheter (i) points to the right. (b) The sheath (i) drags from the left atrial (LA) while the catheter (ii) remains in the LA. (c) The sheath (ii) extends to the LA roof and the catheter (i) makes a circular.

In order to overcome these difficulties, we used some techniques to compensate for the navigation challenges of the RMN catheter in the small LA. The puncture point should be selected in the anterior region of the fossa ovalis, so that the ostium of sheath can be as far away as possible from the PV. For improving the fine movement of the catheter, the position of the sheath can be adjusted during the procedure as follows. First, “point to the light and ablation the right PV,” which means the ostium of sheath points to the opposite direction of the target, while the direction of the magnetic field points to the ablation target (Figure 3(a)). Second, retract the sheath from the LA to the right atrium while the ablation catheter remains in the LA (Figure 3(b)). Third, when the ablation of the inferior PV is performed, the ostium of sheath is extended to the LA roof (Figure 3(c)). The purpose of these maneuvers is to leave enough space for deploying the three magnets of the RMN catheter from the sheath.

The results showed that, after applying the navigation technique above, patients with a small LA had successful electrical isolation of the PV. The results of follow-up showed that the proportion of patients maintained sinus rhythm with small LA that remains high, which was consistent with previous studies [11–13]. However, these techniques significantly increase X-ray time and dose during mapping and ablation. In some centers, the V-drive™ system can be used to robotically move the sheath in the control room without manual maneuvers [14, 15], however, at an increased cost for Chinese patients. The total X-ray time was not different in the two groups because the X-ray time was mainly used in the placement of catheter, transseptal puncture, and PV imaging. These processes “dilute” the differences in X-ray time during mapping and ablation; however, the difference in fluoro time was 37 s and 12 s between Group A and Group B, respectively. The X-ray dose increased in the large LA group because this group had higher body weight, thus needing the increased radiation. The follow-up time was longer in the small LA group, likely due to the proportion of patients maintaining sinus in the small LA group being high, so the patients had better compliance, while some patients in the large LA group often lost follow-up after recurrence.

## 5. Limitations

There are still some limitations in our research. First, although the *in vitro* experiment assessed the flexibility of the catheter, there was no reliable method to measure the contact force of the catheter. Second, the result of *in vitro* test cannot fully represent the situation in LA. Third, the number of patients with nonparoxysmal AF in this study, especially in the large LA group, is limited, which may affect the results.

## 6. Conclusions

AF ablation using RMN is safe and effective. The small LA can bring challenges to navigation using RMN due to only one magnet beyond the distal sheath, thus increasing the need to use X-ray to guide the operator. We recommend that at least two magnets be deployed beyond the sheath tip during mapping and ablation therapy. Despite these navigation challenges, small LA patients had lower AF recurrence rate compared with large LA patients.

## Figures and Tables

**Table 1:** Baseline characteristics of patients.

	Group A (*n*=83)	Group B (*n*=82)	Total (*n*=165)	*P* value
Gender (male/female)	51/32	50/32	101/64	0.951
Age (years)	58.0 ± 11.5	61.1 ± 9.3	60.7 ± 10.9	0.054
Height (cm)	167.5 ± 6.8	167.1 ± 7.9	167.3 ± 7.5	0.652
Weight (kg)	66.5 ± 8.6	72.8 ±18.4	69.6 ± 14.7	0.006
BMI (kg/m^2^)	23.6 ± 2.1	25.6 ± 3.0	24.6 ± 2.8	＜0.001
Paroxysmal/nonparoxysmal AF	71/12	42/40	113/52	＜0.001
Course of AF (month)	36.0 (48.0)	24.0 (60.0)	36.0 (48.0)	0.651
Basic diseases				
** **Hypertension	43	65	108	＜0.001
** **Diabetes	4	6	10	0.729
** **Coronary heart disease	10	15	25	0.263
** **Stroke	13	14	27	0.807
LVEF (%)	63.9 ± 4.2	62.3 ± 4.6	62.8 ± 4.5	0.113
LA diameter (mm)	35.0 ± 3.1	44.2 ± 3.2	39.6 ± 5.6	＜0.001
Four classical pulmonary vein ostia patterns/not	60/23	60/22	120/45	0.899

BMI: body mass index; AF: atrial fibrillation; LVEF: left ventricular ejection fraction. *P* values listed were calculated between Groups A and B.

**Table 2:** Procedural parameters.

	Group A (*n*=83)	Group B (*n*=82)	Total (*n*=165)	*P* Value
Procedure duration (min)	143.9 ± 31.5	139.3 ± 25.1	141.6 ± 28.5	0.295
Duration from mapping begin to ablation finish (min)	100.3 ± 30.1	95.7 ± 25.9	98.0 ± 28.4	0.289
Total X-ray time (sec)	422.0 (228.0)	468.0 (190.0)	432.0 (193.5)	0.399
Total X-ray dose (gy·cm^2^)	17.1 (11.0)	23.9 (19.4)	20.9 (18.4)	<0.001
X-ray time during mapping and ablation (sec)	37.0 (99.0)	12.0 (30.1)	23.0 (58.0)	<0.001
X-ray dose during mapping and ablation (gy·cm^2^)	1.4 (2.7)	0.7 (2.1)	1.0 (2.2)	0.013
Patients using X-ray during mapping and ablation	80	68	148	0.01
Patients adjust sheath during mapping and ablation	47	21	68	<0.001
Patients adjust sheath for several times during mapping and ablation	13	2	15	0.007
Times of adjust sheath during mapping and ablation	1.0 (1.0)	0.0 (1.0)	0.0 (1.0)	<0.001
Heparin	6500.0 ± 1095.4	6073.2 ± 1331.3	5960.6 ± 1334.3	0.283
Complications (minor)	0	0	0	–
Complications (major)	0	0	0	–
LA volume mapped by C3 (ml)	91.8 ± 15.7	129.3 ± 35.6	109.9 ± 35.2	<0.001

C3: Carto 3 system; LA: left atrial. *P* values listed were calculated between Groups A and B.

**Table 3:** Long-term follow-up.

	Group A (*n*=83)	Group B (*n*=82)	Total (*n*=165)	*P* value
Follow-up (month)	27.0 (21.0)	20.0 (18.3)	24.0 (20.0)	0.003

Success of first anatomical attempt				
** **Total	59	47	116	0.065
** **Paroxysmal	52	31	83	0.947
** **Nonparoxysmal AF	7	16	23	0.262

Recurrence				
** **Total	19	34	53	0.009
** **Paroxysmal	13	15	28	0.036
** **Nonparoxysmal AF	6	19	25	0.978

Long-term success (%)				
** **Total	77.1	58.5	67.9	0.009
** **Paroxysmal	81.7	64.3	75.2	0.036
** **Nonparoxysmal AF	50.0	52.5	51.9	0.978

AF: atrial fibrillation. *P* values listed were calculated between normal group and abnormal group.

**Table 4:** Multivariate analysis with logistic regression.

Variables		OR	95% C.I		*P*
			Lower	Upper	
Gender	Male vs. female	1.2	0.4	3.6	0.771
Age (years)	<60 vs. ≥60	0.9	0.3	3.4	0.904
BMI (kg/m^2^)	<24 vs. ≥24	1.9	0.6	6.3	0.301
Paroxysmal AF	Yes vs. No	1.8	0.5	6.7	0.357
LA diameter (mm)	<40 vs. ≥40	7.3	1.7	31.0	0.007
Basic diseases	Yes vs. No	0.8	0.2	4.0	0.815
Four classical pulmonary vein ostia patterns	Yes vs. No	0.5	0.2	1.6	0.234
Procedure duration (min)	<141.6 vs. ≥141.6	3.1	0.6	14.6	0.163
Duration from mapping begin to ablation finish (min)	<98.0 vs. ≥98.0	2.4	0.5	10.7	0.248
Total X-ray time (sec)	<432.0 vs. ≥432.0	0.7	0.2	2.3	0.515

BMI: body mass index; AF: atrial fibrillation; LA: left atrial; OR: odds ratio; CI: confidence interval.

## Data Availability

The figure and table data used to support the findings of this study are included within the article.
